# Risk factors associated with prevalent and incident syphilis among an HIV-infected cohort in Northeast China

**DOI:** 10.1186/s12879-014-0658-1

**Published:** 2014-12-04

**Authors:** Qing-hai Hu, Jun-jie Xu, Hua-chun Zou, Jing Liu, Jing Zhang, Hai-bo Ding, Han-Zhu Qian, Si-ruo Li, Yu Liu, Yong-jun Jiang, Hong Shang, Ning Wang

**Affiliations:** Key Laboratory of AIDS Immunology of National Health and Family Planning Commission, Department of Laboratory Medicine, The First Affiliated Hospital, China Medical University, Shenyang, 110001 P.R. China; Collaborative Innovation Center for Diagnosis and Treatment of Infectious Diseases, Hangzhou, China; School of Public Health and Community Medicine, The University of New South Wales, Sydney, 2052 Australia; Vanderbilt Institute for Global Health, Vanderbilt University, Nashville, TN USA; English Department, University of Tennessee at Chattanooga, Chattanooga, TN USA; National Center for AIDS/STD Control and Prevention, Chinese Center for Disease Control and Prevention, Beijing, China

**Keywords:** Retrospective cohort study, Syphilis, Prevalence, Incidence, Antiretroviral therapy (ART)

## Abstract

**Background:**

Sexually transmitted infections (STIs) increase HIV infectivity through local inflammatory processes. Prevalent and incident STIs among people who live with HIV/AIDS (PLWHA) are indicators of high-risk sexual behaviors and imply potential spread of HIV. Little is known about the prevalence and incidence of concurrent syphilis and associated risk behaviors among PLWHA in China.

**Methods:**

A retrospective cohort study was conducted among PLWHA who attended the outpatient clinic of a designated AIDS treatment hospital in Shenyang, China, between March 2009 and May 2013. Physical examinations and syphilis serology were conducted at each visit. A questionnaire on demographic characteristics was also collected.

**Results:**

A total of 1010 PLWHA were enrolled, of whom 77.0% were men who have sex with men (MSM). The baseline syphilis prevalence among PLWHA was 19.8% (95% confidence interval [CI]:17.3–22.3%). During follow-up, 78.3% retained in the cohort, and contributed a median follow-up of 9.4 months (interquartile range: 5.9-18.7 months). Syphilis incidence among PLWHA was 18.7 (95% CI: 15.5–21.8) per 100 person years. Mulitvariate logistic analysis showed that receiving antiretroviral therapy (ART) (adjusted OR [aOR] = 0.48), older age (≥40 years vs. ≤24 years, aOR = 2.43), being MSM (aOR = 2.30) and having higher baseline HIV viral load (>100000 copies/mL vs. ≤100000 copies/mL, aOR = 1.56) were independent predictors for syphilis infection among PLWHA at enrollment (p < 0.05 for all). Mulivariate Cox regression found that receiving ART (adjusted hazard ratio [aHR] = 1.81), older age (≥40 years vs. ≤24 years, aHR: 5.17) and MSM status (aHR = 2.68) were independent risk factors for syphilis seroconversion (each p < 0.05).

**Conclusions:**

Syphilis prevalence and incidence were high among PLWHA in Shenyang. A campaign focusing on detection and treatment of syphilis among PLWHA is urgently needed, especially one with a focus on MSM who are at a higher risk for syphilis.

**Electronic supplementary material:**

The online version of this article (doi:10.1186/s12879-014-0658-1) contains supplementary material, which is available to authorized users.

## Background

China has witnessed a rapid increase of sexually transmitted infections (STIs), including syphilis and HIV in the past decade [1],[2]. The national sentinel surveillance in 2009 showed that syphilis prevalence was 2.4% among female sex workers (FSWs), 3.4% among people who inject drugs (PWID), and 9.1% among men who have sex with men (MSM) [1]. The population prevalence of syphilis in China was 32.0 per 100,000 people in 2011 [3] which was over 7 times as high as that in the US (4.5 per 100,000 people) [4] and the UK (5.6 per 100,000 people) [5]. In 2008, the prevalence of active syphilis among MSM was 12.2% in China, which was lower than that in Thailand (21.6%) but higher than that in Indonesia (4.0%) [6]. Sexual transmission had become the major route of HIV transmission in China in recent years [7]. Of 70,000 newly reported HIV/AIDS cases in the first nine months of 2013, heterosexual transmission accounted for 69.1% and MSM transmission accounted for 20.8% of all cases [8].

Globally the majority of HIV prevention programs usually focus on HIV negative individuals who are at high risk for HIV infection [9],[10]. However, recently researchers realized that it may be more efficient to prevent HIV if we target people living with HIV/AIDS (PLWHA) (“positive prevention”), as they really serve as the sources of HIV transmission [11]. A meta-analysis showed that positive preventions were efficacious in reducing unprotected sex and acquisition of STIs [12]. The concept of *Treatment as prevention* strategy was raised and investigated quite intensively in recent years, and antiretroviral therapy (ART) can remarkably decrease the risk of transmitting HIV through reducing viral load (VL) among PLWHA [13],[14]. HIV-1 RNA in seminal plasma may decline more slowly than that in blood and could be detected with undetectable HIV-1 RNA in blood plasma when PLWHA experience ART [15],[16], which points to the potential risk for HIV transmission via unprotected sexual behaviors. Moreover, ART may lead to complacent as people feel that they may live a long life with HIV and restore sexual desire as well as physical and immunological functions [17],[18]. Because of sharing a common mode of sexual transmission, syphilis and HIV infection have significant epidemic synergistic. Syphilis incidence is considered an indicator of high-risk sexual behaviors [19],[20]. Therefore, it is useful to understand the risk for HIV transmission among PLWHA by measuring their risk for syphilis transmission [21]. In addition, among individuals diagnosed with HIV who are not yet eligible for ART, treatment of co-infections including syphilis may offer an important alternative approach to reducing HIV VL and decreasing HIV transmission risk [22]. Little is known about the risk for syphilis transmission among PLWHA and its correlation with ART and sexual behaviors [23]. In this retrospective cohort study we aimed to evaluate both syphilis prevalence and incidence among PLWHA in Northeast China.

## Methods

### Study population and participants enrollment

**Figure 1:**
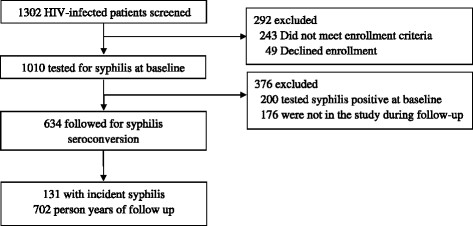
**Procedure of enrollment and follow-up.** A total of 1302 participants were invited, 243 did not meet enrollment criteria, 49 declined enrollment. A total of 1010 participants were included at baseline. Of the 810 participants who were syphilis seronegative at baseline, 634 completed at least one follow-up visit.

This retrospective cohort study was conducted among HIV-infected patients in Shenyang City in Northeast China. HIV status was based on positive test results from two peripheral blood samples by HIV ELISA and confirmed by western blot (Gene labs Diag. Singapore). All HIV testing sites were certified by the National AIDS Reference Laboratory at China CDC. Participants were recruited from The First Affiliated Hospital of China Medical University (CMU), which was one of two designated hospitals providing free ART in Shenyang City. Since 2002, PLWHA in China with a CD_4_^+^ T cell counts of 200 cells/mm^3^ or less, total lymphocyte counts of less than 1200 cells/mm^3^, or WHO disease stage 3 or 4 have been eligible for free combination antiretroviral therapy. In 2008, the CD_4_^+^ T cell counts threshold for treatment was increased to 350 cells/mm^3^ [24]. Between March 2009 and May 2013, patients who were scheduled for a regular visit at the HIV outpatient clinic in The First Affiliated Hospital of CMU were invited to participate in a baseline assessment. Eligible participants were those who: were 18 years or older and were able and willing to provide written informed consent. Baseline syphilis serological test was conducted to assess prevalent syphilis. Syphilis un-infected patients were followed up every 6 months to assess incident syphilis. During the follow-up, we calculated survival as time from baseline initiation to syphilis seroconversion or censoring. Patients were censored at dropout, loss to follow-up, or on May 31, 2013, whichever came first. Those who were diagnosed with syphilis infection were referred to the STI clinic in the same hospital for treatment. Figure 1 shows the study procedures.

Ethical approval was obtained from the institutional review board of The First Affiliated Hospital of CMU prior to the commencement of the study.

### Data collection

At baseline, patients were asked to self-complete an anonymous questionnaire on demographic characteristics, HIV history including date and venues of HIV diagnosis, transmission route, and ART experience. HIV VL and CD_4_^+^ T cell counts were also tested as part of regular care. The blood specimens collected at baseline and 6-monthly follow-up visits were also tested for syphilis infection. After explanation of study, informed consent was obtained from all participants.

### Laboratory testing

Syphilis screening serological test was performed using rapid plasma reagin (RPR; Shanghai Kehua Bio-engineering Co., Ltd, China). Positive RPR results were confirmed using *Treponema Pallidum* particle assay (Serodia TPPA; Fujirebio, Tokyo, Japan). Subjects who were plasma positive for both TPPA and RPR were considered as current infection. RPR negative participants at baseline who turned to be positive for both TPPA and RPR at follow-up visits were defined as syphilis seroconversion event. In addition, TPPA negative participants at baseline who turned to be TPPA positive were eligible for syphilis seroconversion during the follow-up period. CD_4_^+^ T cell counts were determined by flow cytometry (BD Bioscience San Diego, CA, USA). Plasma HIV-1 RNA copy was measured by a commercial HIV RNA quantitative detection assay, COBAS AmpliPrep/COBAS TaqMan HIV-1 Test (Roche, Germany), with a detection limit of 40 copies/mL in plasma. All tests were performed according to the manufacturers’ instructions.

### Data analysis

Demographic and HIV history variables were compared between participants with prevalent syphilis at baseline and those without; and between those who seroconverted during follow-up and those who did not. We analyzed covariates including sex, age (≤24, 25–39, and ≥40 years), ethnicity, marital status, occupation, education, HIV transmission route (heterosexual, MSM and others), HIV diagnosis venues (voluntary counseling and testing [VCT], hospital, others and unknown), baseline CD_4_^+^ T cell counts (<200, 200-349, 350–499, and ≥500 counts/mm^3^), baseline VL (≤100000 and >100000 copies/mL). Logistic regression was performed to assess factors associated with prevalent syphilis infection at baseline. Cox proportional hazard regression was used to determine the adjusted hazard ratio (aHR) for factors associated with incident syphilis infection. Syphilis seroconversion was estimated to occur at the midpoint of the interval between the last negative test and the subsequent follow-up positive test. Syphilis incidence rate and its 95% confidence interval (CI) were calculated assuming Poisson distribution. Variables with *P* < 0.20 in univariate analysis were included in the multivariate logistic or Cox regression models. Only variables with *P* < 0.05 were kept in the final multivariate models. Double data entries were performed using EpiData software (The Epi Data Association Odense, Denmark, version 3.02). Data were analyzed using SAS 9.1 (SAS Institute Inc., Cary, NC).

## Results

### Demographic characteristics of study participants

As shown in Figure 1, a total of 1302 PLWHA who were registered at the HIV clinic were invited to participate in the study, 243 did not meet enrollment criteria, 49 declined enrollment. Therefore a total of 1010 participants were included in the estimation of HIV prevalence at baseline. Of the 810 participants who were syphilis seronegative at baseline, 78.3% (634/810) completed at least one follow-up visit and contributed a median follow-up of 9.4 months (interquartile range [IQR]: 5.9-18.7 months).

Table 1 shows the demographic characteristics and HIV history stratified by syphilis serostatus at baseline and syphilis seroconversion during follow-up. At baseline, the median age was 35 years (IQR: 27-46); 87.4% were ethnic Han; 93.3% were male; 87.6% were Shenyang residents, 34.8% had an education of high school or higher; 51.7% were single, 32.7% were currently married and 15.6% were divorced; 77.0% self-reported as being MSM.

**Table 1:** **Characteristics of PLWHA with prevalent and incident syphilis in Shenyang**

Characteristic	Syphilis infection at baseline			Syphilis seroconversion during follow-up			
	No. of participants	Seropositive cases	Prevalence (%)	No. of participants	Person–years (PY)	Incident cases	Incidence (per 100 PY)
**Overall**	1010	200	19.8	634	702.3	131	18.7
**Gender**							
Female	68	4	5.9	51	40.6	3	7.4
Male	942	196	20.8	583	661.8	128	19.3
**Age (year)**							
Median(IQR)	35(27-46)			35(27-46)			
≤24	142	22	15.5	92	124.8	8	6.4
25-39	461	77	16.7	299	353.7	43	12.2
≥40	407	101	24.8	243	223.9	80	35.7
**Ethnicity**							
Han	883	182	20.6	552	607.0	115	19.0
Other ethnicities	127	18	14.2	82	95.3	16	16.8
**Marital status**							
Single	522	102	19.5	317	376.0	54	14.4
Married	330	62	18.8	213	219.3	44	20.1
Divorced	158	36	22.8	104	107.1	33	30.8
**Occupation**							
Employed	645	129	20.0	415	461.3	90	19.5
Unemployed	198	36	18.2	123	120.0	24	20.0
Retired	76	22	29.0	43	46.5	14	30.1
Student	91	13	14.3	53	74.7	3	4.0
**Education**							
Less than high school	351	77	21.9	209	225.4	46	20.4
High school or more	659	123	18.7	425	476.9	85	17.8
**Shenyang resident**							
Yes	885	181	20.5	560	601.4	115	19.1
No	125	19	15.2	74	100.9	16	15.9
**HIV transmission route**							
Heterosexual	146	16	11.0	103	88.2	15	17.0
MSM	778	171	22.0	478	570.0	111	19.5
Others (injection drug use, blood, unknown)	86	13	15.1	53	44.2	5	11.3
**HIV diagnosis venue**							
VCT	277	54	19.5	182	244.9	41	16.7
Hospital	366	87	23.8	242	281.3	59	21.0
Others (blood donation, physical exam, etc.)	57	6	10.5	48	70.0	5	7.2
Unknown	310	53	17.1	162	106.2	26	24.5
**ART at baseline**							
No	687	159	23.1	385	501.0	68	13.6
Yes	323	41	12.7	249	201.3	63	31.3
**Time since HIV diagnosis (year)**							
≤2	730	156	21.4	439	514.1	79	15.4
>2	280	44	15.7	195	188.2	52	27.6
**Baseline CD**_**4**_^**+**^ **T cell counts(counts/mm** ^**3**^ **)**							
≥500	152	28	18.4	95	126.3	20	21.1
350-499	255	40	15.7	173	194.1	41	23.7
200-349	331	62	18.7	214	226.7	36	16.8
<200	246	65	26.4	142	137.7	30	21.1
Unknown	26	5	19.2	10	17.5	4	40.0
**Baseline VL (copies/mL)**							
≤100,000	599	118	19.7	378	455.6	77	16.9
>100,000	148	46	31.1	72	85.5	13	15.2
Unknown	263	36	13.7	184	161.2	41	25.4

IQR: interquartile range; VCT: voluntary counseling and testing; ART: antiretroviral therapy; VL: viral load; PY: person year.

Just over a quarter (27.4%) of participants had been diagnosed with HIV at VCT venues, 36.2% in hospitals, and the rest through other ways such as HIV/AIDS research projects. Nearly one third (32.0%) had prior ART. At baseline 15.1%, 25.3%, 32.8% and 24.4% had a CD_4_^+^ T cell counts of ≥500, 350-499, 200-349 and <200 cells/mm^3^, respectively. CD_4_^+^ T cells were not detected in 2.6% of participants. At baseline 59.3% and 14.7% had a VL of ≤100,000 and >100,000 copies/mL, respectively. VL was not detected in 26.0% of participants.

### Factors associated with seropositivity of syphilis at baseline

The baseline seroprevalence of syphilis was 19.8% (95% CI: 17.3–22.3%). Table 2 illustrates the factors associated with baseline syphilis infection. In the final multivariate logistic regression model, ART experience was negatively associated with syphilis infection (adjusted OR [aOR] = 0.48, 95% CI: 0.31–0.73), but older age (≥40 years vs. ≤24 years) (aOR = 2.43, 95% CI: 1.42–4.15), being MSM (aOR = 2.30, 95% CI: 1.30–4.08), and having higher baseline VL (>100,000 copies/mL vs. ≤100000 copies/mL) (aOR = 1.56, 95% CI: 1.02–2.40) were independently associated with a higher risk of syphilis infection (each p < 0.05).

**Table 2:** **Factors associated with syphilis infection at baseline among PLWHA in Shenyang**

Factor	OR (95% CI)	***P*** -value	Adjusted OR (95% CI)	***P*** -value
**Gender**				
Female	1.00			
Male	4.20 (1.51 –11.69)	0.006		
**Age(year)**				
≤24 years	1.00			
25-39 years	1.09 (0.65 -1.83)	0.734	1.26 (0.74 –2.12)	0.395
≥40 years	1.80 (1.08 -2.99)	0.023	2.43 (1.42 –4.15)	0.001
**Ethnicity**				
Han	1.00			
Other ethnicities	0.64 (0.38 –1.08)	0.091		
**Marital status**				
Single	1.00			
Married	0.95 (0.67 –1.35)	0.786		
Divorced	1.22 (0.79 –1.87)	0.375		
**Occupation**				
Employed	1.00			
Unemployed	0.89 (0.59 –1.34)	0.573		
Retired	1.63 (0.96 –2.77)	0.072		
Student	0.67 (0.36 –1.24)	0.199		
**Education**				
Less than high school	1.00			
High school or more	0.82 (0.59 –1.12)	0.214		
**Shenyang resident**				
Yes	1.00			
No	0.70 (0.42 –1.17)	0.170		
**HIV transmission route**				
Heterosexual	1.00			
MSM	2.29 (1.33 –3.95)	0.003	2.30 (1.30 –4.08)	0.004
Others (injection drug use, blood, unknown)	1.45 (0.66 –3.18)	0.357	1.33 (0.59 –2.97)	0.488
**HIV Diagnosis venue**				
VCT	1.00			
Hospital	1.29 (0.88 –1.89)	0.195	1.42 (0.94 –2.13)	0.095
Others (blood donation, physical exam, etc.)	0.49 (0.20 –1.19)	0.115	0.63 (0.25 –1.56)	0.314
Unknown	0.85 (0.56 –1.30)	0.453	0.75 (0.49 –1.17)	0.208
**ART at baseline**				
No	1.00			
Yes	0.48 (0.33 –0.70)	<0.001	0.48 (0.31 –0.73)	0.001
**Time since HIV diagnosis (year)**				
≤2	1.00			
> 2	0.69 (0.48 –0.99)	0.044		
**Baseline CD**_**4**_^**+**^ **T counts(counts/ mm** ^**3**^ **)**				
≥500	1.00			
350-499	0.82 (0.48 –1.40)	0.475		
200-349	1.02 (0.62 –1.67)	0.935		
<200	1.59 (0.97 –2.62)	0.068		
Unknown	1.05 (0.37 –3.04)	0.922		
**Baseline VL(copies/mL)**				
≤100000	1.00			
>100000	1.84 (1.23 -2.75)	0.003	1.56 (1.02 –2.40)	0.040
Unknown	0.65 (0.43 -0.97)	0.035	0.82 (0.54 –1.26)	0.361

VCT: voluntary counseling and testing; ART: antiretroviral therapy; VL: viral load.

### Factors associated with seroconversion of syphilis during follow-up

Of the 634 eligible participants for syphilis incidence estimation, 131 (20.7%) seroconverted during follow-up. The incidence rate was 18.7 (95% CI: 15.5–21.8) per 100 person years (PY). Table 3 shows factors associated with syphilis seroconversion. ART experience (adjusted hazard ratio [aHR] = 1.81, 95% CI: 1.25–2.62), older age (≥40 years vs. ≤24 years) (aHR = 5.17, 95% CI: 2.47–10.84), and being MSM (aHR: 2.68, 95% CI: 1.53–4.69) were independently associated with syphilis seroconversion (each p < 0.05).

**Table 3:** **Factors associated with syphilis seroconversion among PLWHA in Shenyang**

Factor	HR (95% CI)	P-value	Adjusted HR (95% CI)	P-value
**Gender**				
Female	1.00			
Male	3.39 (1.08 –10.65)	0.037		
**Age (year)**				
≤24 years	1.00			
25-39 years	1.79 (0.84 -3.81)	0.131	1.86 (0.87 –3.98)	0.108
≥40 years	4.85 (2.34 -10.05)	<0.001	5.17 (2.47 –10.84)	<0.001
**Ethnicity**				
Han	1.00			
Other ethnicities	0.91 (0.54 –1.53)	0.713		
**Marital status**				
Single	1.00			
Married	1.31 (0.88 –1.95)	0.183		
Divorced	2.00 (1.29 –3.08)	0.002		
**Occupation**				
Employed	1.00			
Unemployed	0.98 (0.62 –1.54)	0.924		
Retired	1.58 (0.90 –2.77)	0.112		
Student	0.23 (0.07 –0.72)	0.012		
**Education**				
Less than high school	1.00			
High school or more	0.89 (0.62 –1.27)	0.503		
**Shenyang resident**				
Yes				
No	0.89 (0.53 –1.51)	0.676		
**HIV transmission route**				
Heterosexual	1.00			
MSM	1.45 (0.84 –2.49)	0.181	2.68 (1.53 –4.69)	0.001
Others (injection drug use, blood, unknown)	0.68 (0.25 –1.87)	0.455	0.63 (0.23 –1.75)	0.376
**HIV Diagnosis venue**				
VCT	1.00			
Hospital	2.82 (1.13 -7.03)	0.026	2.08 (0.83 –5.22)	0.120
Others (blood donation, physical exam, etc.)	2.34 (0.92 -5.91)	0.073	1.92 (0.76 –4.87)	0.171
Unknown	3.05 (1.17 -7.98)	0.023	2.55 (0.97 –6.74)	0.058
**ART at baseline**				
No	1.00			
Yes	1.81 (1.27 –2.57)	0.001	1.81 (1.25 –2.62)	0.002
**Time since HIV diagnosis (year)**				
≤2	1.00			
> 2	1.57 (1.10 –2.23)	0.012		
**Baseline CD**_**4**_^**+**^ **T cell counts(counts/mm** ^**3**^ **)**				
≥500	1.00			
350-499	1.26 (0.74 –2.16)	0.392		
200-349	0.92 (0.53 –1.59)	0.764		
<200	1.26 (0.71 –2.22)	0.428		
Unknown	1.83 (0.62 –5.36)	0.272		
**Baseline VL(copies/mL)**				
≤100000	1.00			
>100000	0.97 (0.54 –1.75)	0.920		
Unknown	1.28 (0.87 –1.87)	0.207		

VCT: voluntary counseling and testing; ART: antiretroviral therapy; VL: viral load.

## Discussion

STIs pose considerable health threats to PLWHA. In this study, we report on the first evaluation of both syphilis prevalence and incidence among an HIV-infected cohort in northeast China. The prevalence among PLWHA in Shenyang (19.8%) is much higher than that among PLWHA in Taiwan (5.7%) [25], Guangzhou (10.5%) [26] and Liuzhou City (11.4%) [27] in China. It is also higher than the average syphilis prevalence among PLWHA from a Meta analysis (9.5%) [28]. Syphilis incidence among Shenyang PLWHA (18.7/100 PY) is significantly higher than that in Seoul, South Korea (4.57/100 PY) [29], and higher than the syphilis incidence among Sichuan PWID (4.71/100 PY) [30] and Xichang FSWs (6.23/100 PY) [31].

Syphilis can generate genital ulcers and increase the likelihood of HIV virus shedding. Additionally syphilis co-infection among PLWHA can increase the concentration of HIV RNA in blood plasma and decrease the number of CD_4_^+^ T cell counts [22], and may increase the risk for HIV transmission [32]. It’s highly likely that high prevalent and incident syphilis may contribute to the spread of HIV which is a new threat to HIV prevention. Our findings provide important information to the development of syphilis screening and treatment program for PLWHA across China. Syphilis is a marker of unsafe sexual practices [20],[21], and therefore the high syphilis prevalence and incidence indicated that PLWHA who had already known self HIV positive status may still engage in unprotected sex. Behavioral intervention can reduce HIV transmission risk among HIV positive MSM [33]. Efficient education programs including safer sexual behavioral interventions are urgently needed to reduce risk for STIs transmission among PLWHA in China.

Conflicting results are provided by studies analyzing the association between ART experience and STIs transmission [34]-[36]. Our data show that the syphilis seropositive rate among ART patients was significantly lower than that among treatment naïve patients. The possible reasons for this may include: unrecovered immune status, degenerative sexual desires and lower frequency of unprotected sexual behaviors. Our study further shows that PLWHA who took ART had significantly increased syphilis incidence. This is consistent with the findings by Stolte, et al. in Amsterdam [34], but inconsistent with that by Huang, et al. in Taiwan [37]. Though ART correlate with substantially lowered HIV RNA levels and decreased HIV infectiveness among PLWHA [13],[14], apart from beneficial clinical effects, treatment advances may have unintended effects on sexual behavior. Some evidence suggests that since ART became available, the prevalence of unprotected sex [34],[38] have increased. Sexually transmitted co-infections increase HIV infectiveness through local inflammatory processes and offset the ART treatment effect as being HIV secondary prevention. These findings suggest that, with the expansion of ART in China, health education, behavioral interventions and STIs monitoring should be strengthened among ART patients.

MSM (vs. heterosexual), current use of ART at baseline, and older age (≥40 vs. ≤24 years) were independently correlated with baseline syphilis infection. MSM status is also a risk factor for incident syphilis infection, which is consistent with the literature [36],[39]. In China MSM accounted for 0.3% of the total reported HIV/AIDS cases in 2006. This figure rapidly increased to 13.0% in 2011 [7]. A large number of MSM engage in unprotected sexual behaviors and multiple sexual partnership [40],[41] which place them at risk for both HIV and syphilis infections. Our findings suggest that HIV-infected MSM should be given priority to receive syphilis screening and treatment in Shenyang city.

This study found that the participants aged 40 or older had a significantly higher risk for syphilis than patients aged 24 or younger. HIV infections among the elderly have become increasingly common in China in recent years [7],[42],[43]. The possible reasons for higher risks for HIV and STIs among elderly may include increasing use of commercial sex, infrequent condom use and low uptake of STIs testing [42]-[44].

Our study has limitations. Firstly, we did not collect data on sexual behaviors and therefore couldn’t evaluate the relationship between syphilis and sexual behaviors. Secondly, the participants were a convenience sample of PLWHA who lived in Shenyang city. Results from this study may not represent all individuals who are living with HIV/AIDS in China. Thirdly, higher loss to follow-up is another challenge in our study. By the end of the study period, about 21.7% of the participants were lost to follow-up, and this may cause selection bias. Fourthly, because HIV and syphilis are sensitive topic for participants, this study cannot exclude social desirability bias. Our study, with data on both syphilis prevalence and incidence in HIV-infected population, provides significant evidence for improving HIV/STIs prevention interventions and care, albeit with above-mentioned limitations.

## Conclusions

The prevalence and incidence of concurrent syphilis were both moderately high among PLWHA in Northeast China. Results from this program suggest that receiving ART, older age, and MSM status were independent risk factors for both prevalent and incident syphilis infection. Interventions focusing on the detection and treatment of syphilis are urgently needed for PLWHA in China, especially MSM who are at high risk for syphilis.

## Authors’ original submitted files for images

Below are the links to the authors’ original submitted files for images. Authors’ original file for figure 1
