# Supplementary figures and images for: Risk factors associated with prevalent and incident syphilis among an HIV-infected cohort in Northeast China
Source: BMC Infect Dis. 2014 Dec 4;14:658. doi: 10.1186/s12879-014-0658-1 (PMC4265485; doi:10.1186/s12879-014-0658-1)

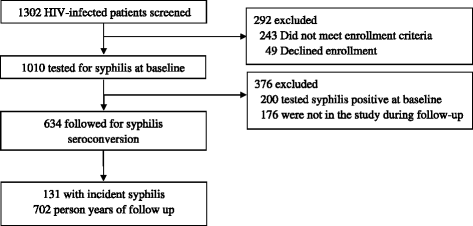

Supplement: Supplementary file 1 — Authors’ original file for figure 1 [file 12879_2014_658_MOESM1_ESM.gif]
